# Necrocide 1 mediates necrotic cell death and immunogenic response in human cancer cells

**DOI:** 10.1038/s41419-023-05740-0

**Published:** 2023-04-05

**Authors:** Jing Zhang, Christina Trojel-Hansen, Jianghuang Wang, Zili Zhang, Xing Wang, Yuhui Qiao, Huike Jiao, Mickaël Michaud, Oliver Kepp, Marja Jäättelä, Guido Kroemer, Qing Zhong

**Affiliations:** 1 Key Laboratory of Cell Differentiation and Apoptosis of National Ministry of Education, Department of Pathophysiology, Shanghai Jiao Tong University School of Medicine, Shanghai, 200025 China; 2 Equipe Labellisée par la Ligue Contre le Cancer, Université de Paris Cité, Sorbonne Université, INSERM UMR1138, Centre de Recherche des Cordeliers, Paris, France; 3 Metabolomics and Cell Biology Platform, Institut Gustave Roussy, 94805 Villejuif, France; 4 Cell Death and Metabolism, Center for Autophagy, Recycling and Disease (CARD), Danish Cancer Society Research Center (DCRC), DK-2100 Copenhagen, Denmark; 5 Department of Cellular and Molecular Medicine, Faculty of Health Sciences, University of Copenhagen, DK-2200 Copenhagen, Denmark; 6 Institut du Cancer Paris CARPEM, Department of Biology, Hôpital Européen Georges Pompidou, AP-HP, 75015 Paris, France

**Keywords:** Cell death, Cancer therapy

## Abstract

Many anticancer agents induce apoptosis, mitotic catastrophe or cellular senescence. Here, we report the functional characterization of an experimental inducer of tumor necrosis factor (TNF)-independent necrosis, necrocide-1 (NC1). NC1 (but not its stereoisomer) killed a panel of human cancer cells (but not normal cells) at nanomolar concentrations and with a non-apoptotic, necrotic morphotype, both in vitro and in vivo. NC1-induced killing was not inhibited by caspase blockers, anti-apoptotic BCL2 overexpression or TNFα neutralization, suggesting that NC1 elicits a bona fide necrotic pathway. However, pharmacological or genetic inhibition of necroptosis, pyroptosis and ferroptosis failed to block NC1-mediated cell death. Instead, NC1 elicited reactive oxygen species (ROS) production by mitochondria, and elimination of mitochondrial DNA, quenching of mitochondrial ROS, as well as blockade of mitochondrial permeability transition with cyclosporine A, interfered with NC1-induced cell death. NC1 induced hallmarks of immunogenic cell death incurring calreticulin (CALR) exposure, ATP secretion and high mobility group box 1 (HMGB1) release. Taken together, these data identify a previously uncharacterized signaling cascade leading to an immunogenic variant of mitochondrion-regulated necrosis, supporting the notion that eliciting regulated necrosis may constitute a valid approach for anticancer therapy.

## Introduction

Anticancer chemotherapeutics have been developed for long in a mostly empiric fashion, by identifying chemical compounds that reduce tumor growth or that kill human cancer cells in vitro [1, 2]. Only recently, so-called targeted agents have been identified in cell-based and molecular screening assays [3–5]. It has been commonly thought that efficient anticancer regimens would either trigger the apoptotic demise of tumor cells [6, 7], to stimulate mitotic catastrophe [8], and/or to induce a permanent arrest in the cell cycle that is generally referred to as “senescence” [9, 10]. Unfortunately, tumor cells develop multiple strategies to evade apoptosis, mostly by upregulating anti-apoptotic proteins or by downregulating pro-apoptotic proteins so that they become increasingly therapy-resistant [7, 11]. Moreover, tumor cells can escape the cell cycle arrest imposed on them by mitotic inhibitors or senescence-inducing drugs [12–14]. This capacity to subvert therapeutic effects may explain why tumors often do not respond or only respond transiently to conventional or targeted anticancer agents.

The recent discovery that necrosis can occur in a regulated fashion and the ever more precise characterization of the underlying molecular mechanisms have spurred great interest [15–17], because non-apoptotic pathways might be instrumental to circumvent the resistance of cancer cells to conventional, pro-apoptotic therapeutic regimens [18–20]. In addition, necrotic cell death has been involved in prominent human diseases including neurodegenerative conditions such as Alzheimer’s and Parkinson’s disease as well as ischemic and infectious disorders [21–23].

Although regulated necrosis has been originally identified as a tumor necrosis factor receptor 1 (TNFR1)-dependent cell death modality, emerging evidence suggests that necrosis can also occur in a regulated fashion in response to other stimuli including alkylating DNA damage, reactive oxygen species (ROS) overgeneration and calcium overload [24–27]. Recently, several distinct forms of programmed cell death (PCD) have been defined with unique cellular mechanisms. An important role downstream of the RIPK1-RIPK3-containining complex leading to necroptosis has been highlighted for the mixed lineage kinase like (MLKL) protein [28–31]. Gasdermins have been shown to form pores that cause pyroptosis after cleavage by inflammatory caspases including caspase 1, 4, 5 and 11 [32, 33]. The research field of ferroptosis, driven by iron-dependent phospholipid peroxidation, and regulated by multiple cellular metabolic pathways including redox homeostasis, iron handling, mitochondrial activity and metabolism of amino acids and lipids, has seen exponential growth over the past few years since the term was coined in 2012 [24, 34]. Intriguingly, therapy-resistant cancer cells, particularly those in the mesenchymal state and prone to metastasis, are exquisitely vulnerable to ferroptosis.

Here, we report the identification of a small molecule compound from an optimized derivative of a previous reported anti-tumor compound [35, 36], with its involvement in necrotic cell death never described. 3-cycloheptyl-3-(4-hydroxyphenyl)6-methoxy-7-methyl-1,3-dihydroindole-2-one (which we named necrocide 1, NC1) operates as a potent inducer of regulated necrosis distinct from necroptosis, ferroptosis and pyroptosis, in vitro and in vivo. We characterized the signaling pathways underlying the pro-necrotic activity of NC1.

## Results and discussion

### NC1 induces necrosis in vitro and inhibits tumor growth in vivo

**Fig. 1:**
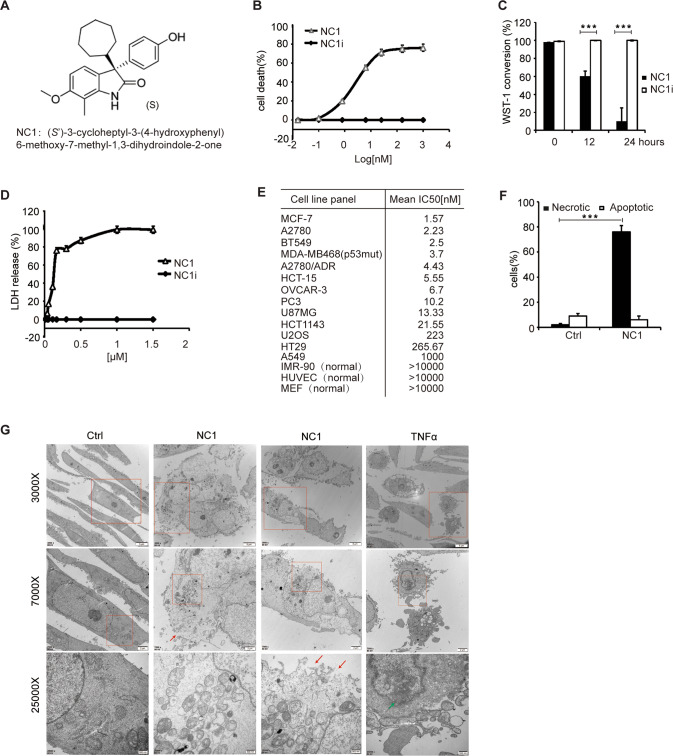
NC1 induces regulated necrosis in human cancer cells. **A** Chemical structure of necrocide 1 (NC1, (*S*’)-3-cycloheptyl-3-(4-hydroxyphenyl)6-methoxy-7-methyl-1,3-dihydroindole-2-one, the active stereoisomer). **B** Dose-dependent killing of MCF-7 breast cancer cells by NC1 but not by its inactive stereoisomer (NC1i). Conversion of the tetrazolium salt WST1 was measured by colorimetry 24 h after the addition of the indicated concentrations of either NC1 or NC1i to measure cell death. **C** Time-dependent killing of MCF-7 cells by NC1 but not by NC1i, as determined 12 or 24 h after the exposure of cells to 10 nM NC1 by WST1 conversion assays. **D** Dose-dependent killing of MCF-7 cells by NC1 but not by NC1i, as determined by the release of lactate dehydrogenase (LDH) into the culture supernatant. **E** IC_50_ values of NC1 on a panel of different human cancer cell lines, normal cells and mouse embryonic fibroblast (MEF), as determined by WST1 conversion assay 24 h after addition of the compound to the cells. **F**, **G** Effects of NC1 on cellular ultrastructure. MCF-7 cells exposed to NC1 were examined by transmission electron microscopy (scale bars = 500 nm/2 µm/5 µm as indicated). Red arrows indicate membrane rupture. Green arrows indicate chromatin condensation. Percentage of MCF-7 cells with an apoptotic or necrotic appearance as shown in (**F**). Values are means ± standard deviation of *n* = 3 independent determinations. Asterisks indicate significance between the two groups indicated (*p < 0.05; **p < 0.01, **p < 0.001).

(*S*’)-3-cycloheptyl-3-(4-hydroxyphenyl)6-methoxy-7-methyl-1,3-dihydroindole-2-one (NC1) represents the third-generation of small molecule compound optimized from earlier generations that mediate robust anti-tumor activity against a variety of cancer cells and mouse tumor xenografts through unknown mechanism [35, 36]. The active NC1 stereoisomer (Fig. 1A), but not its inactive stereoisomer (NC1i, R form), reduced the metabolic activity of human breast carcinoma MCF-7 cells in a dose- and time-dependent fashion (Fig. 1B, C), as it induced cell killing indicated by the release of lactate dehydrogenase into the culture medium (Fig. 1D). Tetrazolium conversion assays revealed the IC_50_ of NC1 to be below 15 nM for a series of human cancer cell lines tested. In contrast, NC1 failed to kill non-transformed IMR-90 fibroblasts, human umbilical vein endothelial cells (HUVECs) and mouse embryonic fibroblast (MEF), even at a concentration of 10 µM (Fig. 1E).

NC1 caused ballooning of MCF-7 cells, which became translucent in phase contrast microscopy, accompanied by an increase in nuclear diameter as well as by the formation of Hoechst 33342-positive chromatin clumps adjacent to the nuclear envelope (Supplementary Fig. 1A, B). Transmission electron microscopy confirmed the necrotic appearance of NC1-treated cells, manifesting a prominent swelling of cytoplasmic organelles, extranuclear vacuolization, and plasma membrane rupture (Fig. 1F, G).

Cytochrome *c* and cathepsin L, which are usually sequestered within mitochondria or lysosomes, respectively, diffusely distributed throughout the cytoplasm upon NC1 treatment, as assessed by immunofluorescence microscopy upon staining with specific antibodies (Supplementary Fig. 1C–F). Thus, we hypothesized that NC1 would induce both mitochondrial membrane permeabilization (MMP) and lysosomal membrane permeabilization (LMP). MMP was confirmed by staining NC1-treated MCF-7 cells with the mitochondrial transmembrane potential (Δψ_m_)-sensitive fluorophore tetramethyl rhodamine methylester (TMRM). LMP was corroborated on MCF-7 cells succumbing to NC1 by means of Lysotracker red staining. Thus, NC1 caused a drastic time-dependent reduction in Δψ_m_ and lysosomal membrane integrity (Supplementary Fig. 1E). Altogether, these results indicate that NC1 stimulates the necrotic demise of human cancer cells.

**Fig. 2:**
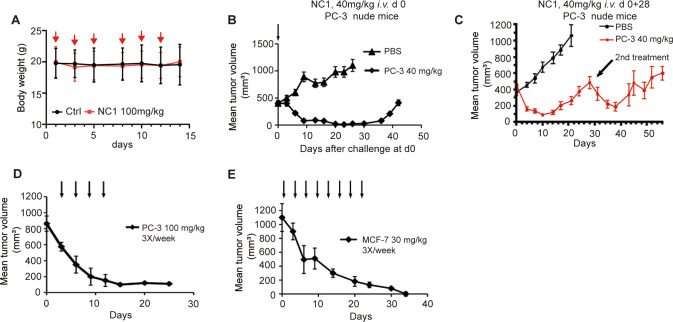
NC1 is a potent anti-tumor agent in vivo. **A** When the tumor volume reached about 200 mm^3^, MCF-7 xenografted nude mice were given NC1 (100 mg/kg) by gavage three times a week for 2 weeks (*n* = 13 in each group). The body weight of mice was measured at each time of administration. **B** Single dose administration of NC1 (40 mg/kg, *i.v.*) causes sustained tumor regression in mouse *xenografts* of PC-3 cells (*n* = 10 per group). **C** Nude PC-3 tumor bearing mice were given one single *i.v.* injection at 40 mg/kg at day 0 followed by a second *i.v.* injection at 40 mg/kg at day 28 to monitor the tumor growth (*n* = 10 per group). Athymic (*nu/nu*) mice were inoculated subcutaneously with PC-3 (**D**) or MCF-7 cells (**E**) and tumor growth (means ± SEM, *n* = 10 mice per group) was monitored after oral administration of 100 mg/kg NC1 (**D**) or intravenous administration of 30 mg/kg NC1 (**E**) (as indicated by arrows).

In the next step, we investigated the tumor-suppressive effects of NC1 in vivo utilizing human cancers xeno-transplanted into immunodeficient *nu/nu* mice. Importantly, NC1 was in general well tolerated by mice. Body weight reduction or other gross pathological signs were not observed following multiple administration schedules at the dose of 100 mg/kg (Fig. 2A). Significant tumor-regression could be induced in PC-3 xenografts by one single intravenous (*i.v.*) injection of NC1 at 40 mg/kg (Fig. 2B). Tumor-regression was sustained up to 20 days before re-growth was observed. The relapsed tumors remained sensitive to a second *i.v.* administration of NC1, which again induced sustained tumor-regression (Fig. 2C). In additional experiments, NC1 treatment was initiated at large tumor sizes (around 1000 mm^3^) by oral gavage (100 mg/kg, 3 times/week). This protocol also consistently reduced the tumor growth of PC-3 xenografts (Fig. 2D). Additionally, continuous administration of NC1 successfully suppressed tumors in mice bearing xenografts of human MCF-7 breast cancers (Fig. 2E). Histochemical analyses of NC1-treated tumors revealed a marked induction of necrosis, in contrast to tumors from saline-treated animals (Supplementary Fig. 2A). Altogether, we conclude that NC1 can induce cell death of human cancer xenografts in vivo.

### Molecular mechanisms of NC1-induced necrosis

**Fig. 3:**
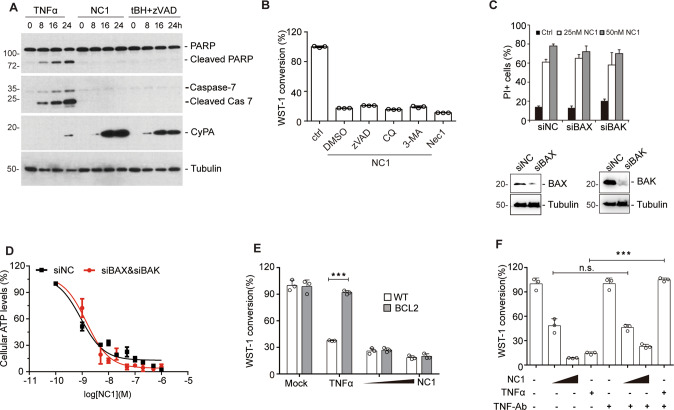
NC1 elicits a non-apoptotic cell death independent of TNFR1 pathway. **A** Lack of caspase activation by NC1. MCF-7 cells were lysed at the indicated time after the addition of TNFα or NC1, and lysates were subjected to immunoblotting for the detection of poly (ADP-ribose) polymerase 1 (PARP1) and proteolytically mature caspase-7. Supernatant was collected to detect the release of Cyclophilin A (CyPA) into the extracellular medium. Tubulin was detected to ensure equal lane loading. **B** MCF-7 cells were treated with NC1 and(or) apoptosis inhibitor z-VAD-fmk, necrosis inhibitor Necrostain-1 (Nec1), autophagy inhibitor chloroquine (CQ) and 3-MA. Cell viability was determined by measuring cellular ATP levels. The data are represented as the mean ± SD of triplicate wells. **C** MCF-7 cells were transfected with siRNA specially against BAX or BAK for 48 h, then subjected to 25 nM NC1 for 12 h to detect cellular viability. **D** MCF-7 cells were transfected with siRNA specific for BAX and BAK for 48 h, then subjected to the indicated concentration of NC1 for 12 h before detecting cellular viability. **E** Failure of BCL2 to inhibit NC1-induced cell killing. Parental or BCL2-overexpressing MCF-7 cells were exposed for 24 h to TNFα or NC1, and WST1 conversion was assessed to estimate cellular viability. **F** Putative contribution of TNFα to NC1-induced cell killing. MCF-7 cells were treated for 24 h with the indicated combinations of NC1, TNFα and TNFα neutralization antibody (TNF-Ab), followed by measurement of cellular ATP level. Asterisks indicate significance between the two groups indicated (*p < 0.05; **p < 0.01, **p < 0.001).

Next, we aimed at further characterizing the necrotic cell death subroutine triggered by NC1. Live cell imaging analysis demonstrated the ballooning of MCF-7 cells upon NC1 treatment (Supplementary Fig. 3A and Movie 1). Utilizing Sytox Green, a dye that only stains dead cells (because it penetrates the permeabilized plasma membrane) but is excluded by live cells, we successfully visualized the dramatic ballooning of NC1-treated cells that occurs during or shortly before cell death. These data suggest MCF-7 cells upon NC1 treatment likely undergo necrosis rather than apoptosis. Contrarily to TNFα induced caspase activation, NC1 failed to activate the cleavage of caspase-7 or Poly (ADP-ribose) polymerase 1 (PARP1), that would occur during the execution of apoptosis in MCF-7 cells (Fig. 3A). In accordance with a paper reporting its role as a necrotic marker [37], Cyclophilin A (CyPA) was released into the extracellular medium when cells were treated with tert-butyl hydroperoxide (tBH) in the presence of z-VAD-fmk to induce necrosis. Consistently, we detected CyPA release at an early stage of NC1 treatment (Fig. 3A). The broad-spectrum caspase inhibitor Z-VAD-fmk, the autophagy inhibitors chloroquine (CQ) and 3-methyladenine (3-MA), as well as the necroptosis inhibitor necrostatin-1 (Nec1) targeting receptor-interacting serine/threonine-protein kinase 1 (RIPK1), all failed to inhibit NC1-induced cell death, suggesting that NC1-induced necrosis differs from apoptosis, autophagic cell death and necroptosis (Fig. 3B). Genetic depletion of the pro-apoptotic factors BAX and BAK by specific siRNAs was unable to rescue NC1-induced cell death (Fig. 3C, D). While the transfection-enforced overexpression of the anti-apoptotic protein BCL2 successfully prevented TNFα-induced apoptosis, it failed to protect cells from NC1-induced killing (Fig. 3E). Along similar lines, TNFα neutralization antibody failed to prevent NC1 induced necrosis in MCF-7 cells (Fig. 3F), suggesting that NC1-induced cell death is independent of the TNF signaling pathway. Altogether, these data demonstrate that NC1-mediated cell death is different from apoptosis.

Several regulated non-apoptotic pathways have been recently characterized, including necroptosis, ferroptosis and pyroptosis. Necroptosis requires the activation and translocation of receptor-interacting serine/threonine-protein kinases RIPK1 and RIPK3 from the plasma membrane to cytosol [28–30, 38], where Mixed lineage kinase like (MLKL) protein is phosphorylated by the RIPK1-RIPK3 complex and activated to execute necrosis in a caspase independent manner [25, 31]. Ferroptosis is characterized by iron-dependent accumulation of oxidized polyunsaturated fatty acid-containing phospholipids on the plasma membrane, which is negatively regulated by the phospholipid hydroperoxide-reducing enzyme glutathione peroxidase 4 (GPX4) and coenzyme Q10 catalyzing enzyme ferroptosis suppressor protein 1 (FSP1) in parallel systems. Pyroptosis is marked by activation of inflammation-activated caspase-1 and lipopolysaccharide (LPS)-activated caspase-11/4/5 [39, 40] and cleavage of pyroptosis executioner, the pore-forming protein gasdermin D (GSDMD) [32, 33].

**Fig. 4:**
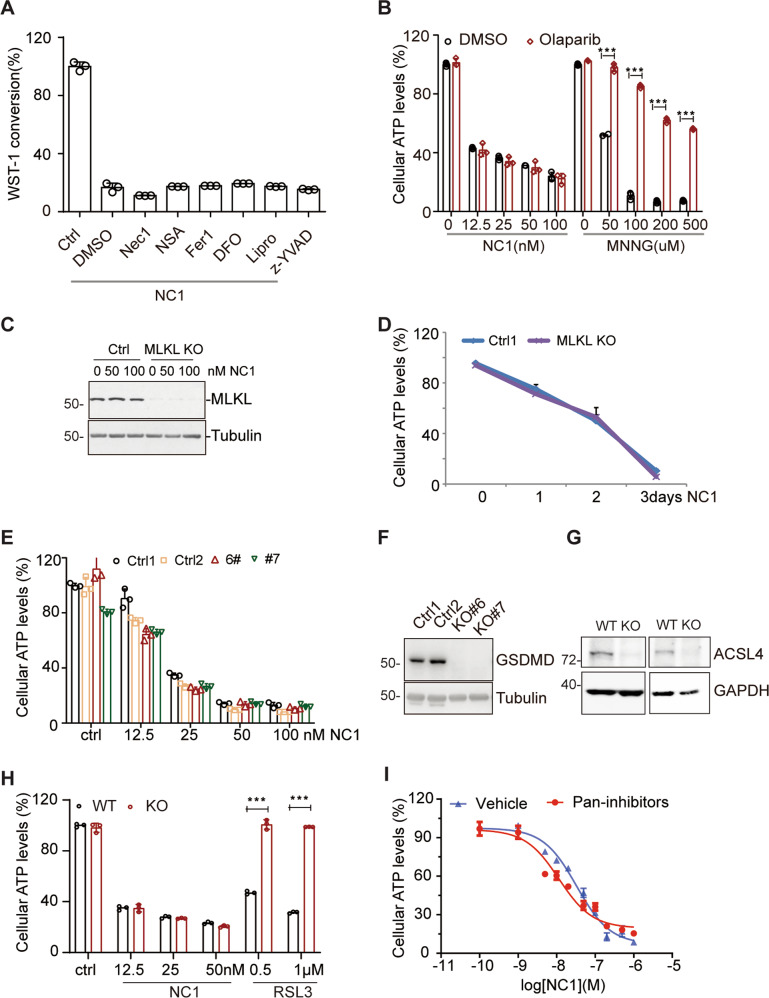
NC1-induced cell death is different from necroptosis, pyroptosis and ferroptosis. **A** MCF-7 cells were treated with NC1 and (or) necroptosis inhibitor Nec-1 and NSA, ferroptosis inhibitor ferrostatin-1 (Fer1) and the iron chelator deferoxamine (DFO), Liproxstatin-1 (Lipro), pyroptosis inhibitor z-YVAD-fmk. Cell viability was determined by measuring cellular ATP levels. **B** MDA-MB-468 cells were treated with indicated concentrations of NC1 or MNNG, in the presence or absence of Olaparib for cell viability analysis. **C**, **D** MLKL was knocked out in MCF-7 cells by CRISPR, then treated by NC1 (50 nM) for different time as indicated. Expression of MLKL was detected by western blotting in MCF-7 control and MLKL knockout cells (**C**). Cell viability was determined by measuring cellular ATP levels by Cell Titer Glo (**D**). **E**, **F** GSDMD was knocked out in MDA-MB-468 cells by CRISPR, then subjected to different concentrations of NC1 treatments for 48 h to measure cell viability (**E**). The expression of GASMD was detected by immunoblotting (**F**). **G**, **H** ACSL4 was knocked out in MDA-MB-468 cells by CRISPR (**G**), then subjected to different concentrations of NC1 or RSL3 treatments overnight to measure cell viability (**H**). **I** MCF-7 cells were precultured with pan-inhibitors (combined treatments of NSA, zVAD-fmk and Z-YAD-fmk, 20 μM each) for 24 h, followed by indicated concentrations of NC1 for overnight. Cell viability was determined by measuring cellular ATP levels. The data are represented as the mean ± SD of triplicate wells. Asterisks indicate significance between the two groups indicated (*p < 0.05; **p < 0.01, **p < 0.001).

We used chemical approaches to define NC1 induced necrosis. The necroptosis inhibitors necrosulfonamide (NSA) and necrostatin-1 (Nec1), the ferroptosis inhibitor liproxstatin-1 (Lipro) and desferrioxamine (DFO), as well as the pyroptosis inhibitor z-YVAD all failed to inhibit NC1-induced cell death (Fig. 4A), indicating that NC1-induced necrosis is different from necroptosis, ferroptosis and pyroptosis. In addition, the PARP inhibitor olaparib failed to inhibit NC1-induced cell death, while it effectively blocked MNNG-mediated parthanatos (Fig. 4B). Suppression of the necroptosis effector MLKL by CRISPR/Cas9 was unable to antagonize NC1 induced cell death, as demonstrated by monitoring cell viability (Fig. 4C, D). Loss of the pyroptosis effector GSDMD also did not block NC1-induced cell death either (Fig. 4E, F). Moreover, suppression of ACSL4 failed to block NC1-induced cell death, although ACSL4 deficiency dramatically rescued RSL3-induced ferroptosis, as reported (Fig. 4G, H). To rule out the implication of complex death forms such as panoptosis, we used combined inhibitions of different cell death pathways including NSA, zVAD-fmk and z-YVAD. Still, the combined inhibition of complex cell death pathways did not affect NC1-induced cell death (Fig. 4I). Taken together, these data suggest that NC1-induced necrosis represents a previous undescribed cell death pathway other than apoptosis, autophagy, necroptosis, ferroptosis, pyroptosis and parthanatos.

### Mitochondria are involved in NC1-induced necrosis

**Fig. 5:**
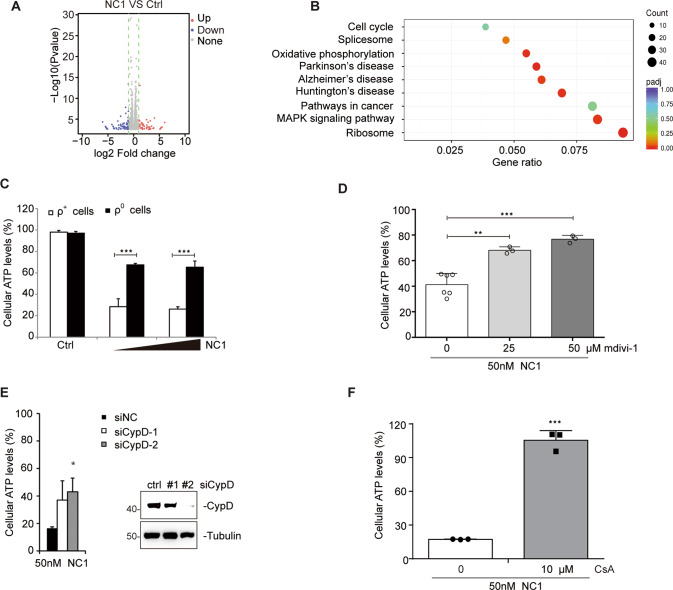
Mitochondria is crucial for NC1-induced cell death. **A** Volcano plot of upregulated or downregulated genes in MCF-7 cells subjected to NC1 treatment. **B** The Ingenuity Pathway Analysis (IPA) of RNA-seq. **C** The mitochondria-depleted MCF-7 cells as well as the control cells were treated with NC1. Cell viability was determined by measuring cellular ATP levels. **D** MCF-7 cells were pre-treated by mitochondrial division inhibitor mdivi-1 (0, 25, 50 μM) for 2 h, followed by NC1 (50 nM) for overnight to measure cellular ATP levels. **E** Inhibition of NC1-induced killing by knockdown of cyclophilin D (CypD). Cells were subjected to the knockdown of CypD using suitable siRNAs. Forty-eight hours post-transfection the cells were treated with NC1 (50 nM, left) to measure cellular ATP levels, and the efficacy of the knockdown was determined by immunoblot (right). **F** MCF-7 cells were pre-treated by mitochondrial permeability transition pore (mPTP) inhibitor cyclosporin A (CsA, 5 μM) for 2 h, followed by NC1 (50 nM) treatment for overnight to measure cell viability. Cell viability was determined by measuring cellular ATP levels. The data are represented as the mean ± SD of triplicate assessments. Asterisks indicate significance between the two groups indicated (*p < 0.05; **p < 0.01, **p < 0.001).

To decipher the mechanism of NC1-induced necrosis, RNA sequencing (RNA-seq) was executed. Bioinformatic analysis of RNA-seq data led to the conclusion that a large proportion of the altered genes were important for oxidative phosphorylation (Fig. 5A, B). The mitochondrial transmembrane potential (∆Ψm) was examined during NC1-induced necrosis and a loss of ∆Ψm actually occurred before plasma membrane rupture (Supplementary Fig. 4A), suggesting that mitochondrial permeability transition (MPT) is involved in NC1-induced necrosis. Therefore, we next investigated if mitochondria participate in NC1-induced necrosis, we generated mitochondrial DNA-depleted cells (ρ° cells) as previously reported [32] and observed that ρ° cells were resistant to NC1-induced necrosis (Fig. 5C). Mitochondrial fusion and division play important roles in cell death. The most efficacious fission inhibitor, mdivi-1 (for mitochondrial division inhibitor) attenuates mitochondrial division in yeast and mammalian cells by acting on dynamin [41]. Mdivi-1 partially suppressed the cytotoxicity of NC1, further supporting the involvement of mitochondria in NC1-induced necrosis (Fig. 5D). Cyclophilin D (CypD) is required for opening of the mitochondrial permeability transition pore (mPTP) [42, 43]. Importantly, knockdown of CypD blocked NC1-induced killing (Fig. 5E). Moreover, cyclosporin A (CsA), which attenuates mitochondrial permeability transition by pharmacologically inhibiting CypD [44], dramatically blocked NC1-induced cell death (Fig. 5F). Together, these data suggest that NC1 drives mitochondria regulated necrosis.

**Fig. 6:**
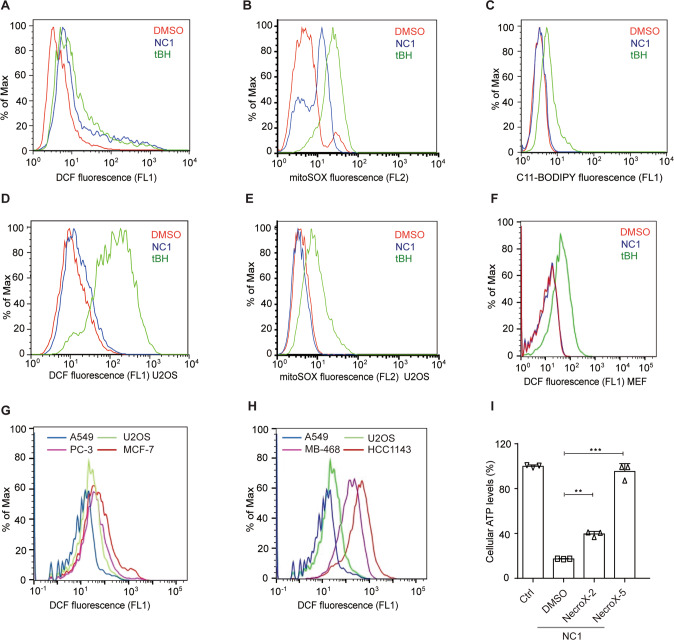
High basal ROS levels in the cancer cell lines correlate with their sensitivity to NC1-induced cell death. MCF-7 cells were treated with DMSO, indicated concentrations of NC1 (50 nM) or tert-butyl hydroperoxide (tBH, 100 μM) for 4 h, cytosolic, mitochondria and lipid ROS production assessed by flow cytometry using H2DCFDA (**A**), MitoSox (**B**) and C11-BODIPY (**C**) respectively. Cytosolic and mitochondria ROS production assessed over time (4 h) by flow cytometry using H2DCFDA (**D**) and MitoSox (**E**) in U2OS cells, or H2DCFDA (**F**) in MEF cells. **G**, **H** Cytosolic ROS production assessed over time (4 h) by flow cytometry using H2DCFDA in sensitive PC-3, MCF-7, MDA-MB-468, HCC1143 cell lines and insensitive A549 and U2OS cells. **I** MCF-7 cells were treated with NC1 and (or) necrosis inhibitor with antioxidant activity NecroX-2 and NecroX-5. Cell viability was determined by measuring cellular ATP levels. The data are represented as the mean ± SD of triplicate wells. Asterisks indicate significance between the two groups indicated (*p < 0.05; **p < 0.01, **p < 0.001).

NC1 provoked a rapid increase in the production of ROS in MCF-7 cells, as detected by flow cytometry upon staining with the ROS-sensitive dye 2’,7’-dichlorofluorescein-diacetate (DCFH-DA) (Fig. 6A). As a positive control for ROS induction we used, tert-butyl hydroperoxide (tBH), an alkyl hydroperoxide. NC1 increased the signal measured by the ROS probe MitoSOX (which detects ROS at mitochondria) but not that one measured by C11- BODIPY (which detects lipid peroxidation) (Fig. 6B, C). In contrast to MCF-7 cells which are killed by NC1 and produced ROS, NC1-insensitive U2OS and MEF cells did not produce DCFH-DA- and MitoSOX-detectable ROS upon NC1 exposure, suggesting a correlation between ROS production and sensitivity to NC1 (Fig. 6D–F). Indeed, four cell lines including PC-3 prostate cancer cells and three human breast cancer cell lines (MDA-MB-468, MCF-7 and HCC1143), which are all highly sensitive to NC1, exhibited high ROS production at baseline. In contrast, basal ROS levels were much lower in NC1-resistant A549 lung cancer cells and U2OS osteosarcoma cells (Fig. 6G, H). These data suggest that high basal ROS levels predispose to NC1-induced cancer cell killing. Accordingly, two necrosis inhibitors (NecroX-2 and NecroX-5) that scavenge mitochondrial ROS blunted NC1 cytotoxicity (Fig. 6I). All these data suggested that mitochondrial ROS production is involved in NC1-induced necrosis.

### Immunogenic cell death induced by NC1

**Fig. 7:**
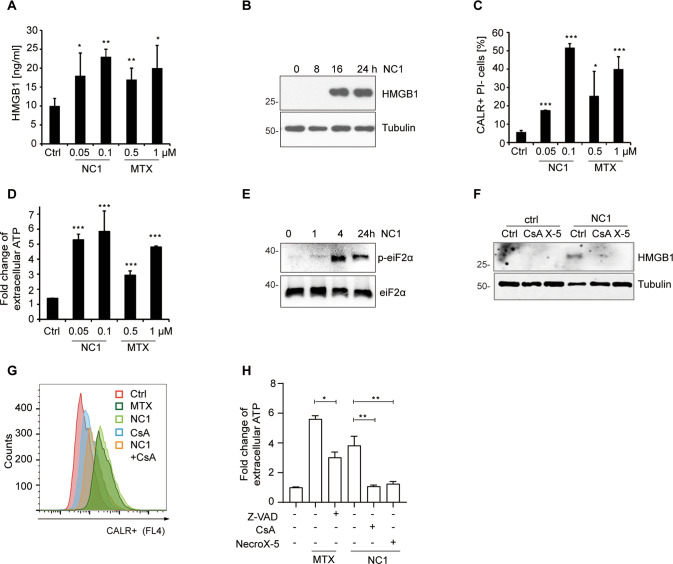
NC1-induced immunogenic cell death (ICD). Induction of the hallmarks of ICD in MCF-7 cells treated with NC1 or mitoxanthrone (MTX) for 48 h, followed by the release of ELISA-detectable HMGB1 (**A**), immunoblotting detection of HMGB1 in the supernatant of untreated control (Ctrl) or NC1(100 nM)-treated cells by indicated times (**B**), cytofluorometric analysis of calreticulin (CALR) exposure on viable (PI^−^) cells (**C**) or the release of ATP into the supernatant by means of a luciferase-based assay (**D**). Results are shown as means ± SD of triplicates, and asterisks indicate significant (*p* < 0.05) effects as compared to untreated controls (Ctrl). **E** Induction of eIF2α phosphorylation by NC1, as determined by immunoblot. **F**–**H** Impact of CsA, NecroX-5 (X-5) or Z-VAD-fmk on ICD hallmarks induced by NC1 or MTX. MCF-7 cells were treated with the indicated combinations of drugs, and HMGB1 release (**F**), calreticulin exposure (**G**) and ATP release (**H**) and were measured. Results are shown as means ± SD of triplicates and are representative of at least three experiments. Asterisks indicate significance compared with control group, or otherwise the two groups indicated (*p < 0.05; **p < 0.01, **p < 0.001). All experiments have been repeated at least three times with similar results.

Immunogenic cell death (ICD) induced by anthracyclines (such as mitoxantrone, MTX) is characterized by the exposure of calreticulin (CALR) on the cell surface, the secretion of ATP during the blebbing phase of apoptosis, as well as the cellular release of HMGB1 from the nucleus and the permeabilized plasma membrane [45]. NC1 was capable of inducing these hallmarks on MCF-7 breast cancer cells as efficiently as did MTX (Fig. 7A–D). This phenomenon was accompanied by an endoplasmic reticulum stress response (as illustrated by the phosphorylation of the eukaryotic translation initiation factor, eIF2α) (Fig. 7E). NC1 and MTX both were equivalent in inducing the ICD hallmarks. Of note, the NC1-induced CALR exposure, ATP and HMGB1 release were inhibited by CsA and necroX-5 (Fig. 7F–H). Altogether, these results suggest that NC1 can induce a type of mitochondria regulated necrosis that bears biochemical and functional hallmarks of ICD.

## Discussion

In this paper, we report the characterization of a small molecular compound, (*S*’)-3-cycloheptyl-3-(4-hydroxyphenyl)6-methoxy-7-methyl-1,3-dihydroindole-2-one (NC1), which kills a panel of human cancer cells by inducing regulated necrosis. The necrotic demise induced by NC1 is distinct from necroptosis in thus far that it neither depends on TNFR1 signaling nor requires caspase-8 inhibition. Characterization of NC1-elicited signals point to the involvement of mitochondria through a non-conventional pathway leading to regulated necrosis. The robust anti-tumor effects of NC1 suggest that the induction of regulated necrosis holds promising potential for the development of future anticancer therapies.

Resisting cell death, especially apoptosis is a hallmark of cancer. One mechanism by which tumor cells develop resistance to cytotoxic agents and radiation is related to disabling of the pathways leading to apoptosis. Our results presented here indicate that NC1 does not activate apoptotic signaling pathways, but directly induces necrotic cell death in a series of human cancer cells. NC1 fails to induce the hallmarks of apoptosis (cellular and nuclear shrinkage with global chromatin condensation) and rather triggers the morphological characteristics of necrosis (oncosis, rather partial peripheral chromatin condensation without nuclear pyknosis, plasma membrane rupture without preceding blebbing) [46–48]. At the functional level, NC1 fails to activate caspases and kills cells in a fashion that is not perturbed by the addition of caspase inhibitors, nor modulated by changes in the expression level of prominent multidomain proteins from the Bcl-2 family including BCL2 itself, BAX and BAK. It is worth mentioning that NC1 also kills TP53-mutant MDA-MB-468 cells [49], therefore it will be interesting to explore to which extent NC1 acts in a TP53-independent fashion.

Several effectors of necrotic cell death have been reported. The phosphorylated MLKL binds to phosphatidylinositol lipids, moves from the cytosol to the plasma and intracellular membranes and directly disrupts membrane integrity, resulting in necroptosis [50]. Gasdermin D (GSDMD), a gasdermin-family member, is cleaved by both caspase-1 and caspase-11/4/5 to release its gasdermin-N domain that perforates the plasma membrane to induce cell swelling and pyroptosis. However, neither suppression of MLKL nor GSDMD blocked NC1-induced cell death. Ferroptosis is usually accompanied by a large amount of iron accumulation and lipid peroxidation during the cell death process. However, we were unable to detect lipid peroxidation upon NC1 treatment. Moreover, the iron chelator DFO, which inhibits ferroptosis, failed to block NC1-induced cell death. However, our data demonstrated that mitochondria play a critical role in NC1 induced cell death. Knockdown of cyclophilin D, which is required for the pro-necrotic permeabilization of mitochondrial membranes, confers partial protection against NC1-mediated cell killing. Moreover, CsA, which attenuates mitochondrial permeability transition, strongly inhibited NC1-induced cell death. Together, these data suggest the obligate contribution of mitochondria to the lethal signaling cascade triggered by NC1.

In summary, NC1 induces necrotic cell death in a series of cancer cell lines. Moreover, NC1 induces therapeutic necrosis of human cancer xenografts in vivo. For its cytotoxic activity, NC1 did not require effector molecules involved in apoptosis, ferroptosis, necroptosis or pyroptosis, but did involve mitochondrial permeability transition and ROS production. In sum, our results support the notion that the induction of programmed necrosis may constitute a future approach for anticancer therapy.

## Materials and methods

### Reagents

The necrocide 1 (TP202377) compound was synthesized as described previously [35]. The following reagents are purchased from companies: Hematoxylin, eosin, Acridine Orange and Hoechst 33342 (Sigma). Sytox Green (Invitrogen). z-VAD-fmk (ApexBio). Necrostatin-1, Necrostatin-5, Ferrostatin-1, Desferrioxamine (DFO), 3-MA, α-Tocopherol (Sigma), NecroX-2 and NecroX-5 (Enzo Life Sciences). Human recombinant TNF-α (R&D Systems). ENLITEN ATP assay kit (Promega). The following antibodies were used: PARP (Cell Signaling Technology, 9542), caspase-7 (Cell Signaling Technology, 9746), CyPA (Santa Cruz), HMGB1 (Cell Signaling Technology, 3935), MLKL (Novus Biologicals, NBP3-03885), GSDMD (Thermo Fisher), beta tubulin (Sigma, T2200), TNF neutralizing antibody (R&D Systems), Cathepsin L (BD Transduction Laboratories, 611084). Full and uncropped western blots were uploaded in the Supplementary Material.

### Cell culture

All cell lines were obtained from ATCC (ATCC/LGC Promochem, Borås, Sweden). Cell culture reagents were obtained from Invitrogen except otherwise stated. All cell lines were maintained according to American Type Culture Collection guidelines in Dulbecco’s modified Eagle’s medium (DMEM, Sigma) containing 10% Fetal Bovine Serum (FBS) 100 U/ml penicillin, and 100 mg/ml streptomycin. For experiments, cells were trypsinized and seeded in dishes (Santa Cruz) and allowed to grow to reach 70% confluence at 37 °C with 95%/5% air/CO_2_ and 80% relative humidity. Cells were treated with compound for indicated concentrations and periods (necrocide 1, TNFα (20 ng/ml), TNF neutralization antibody (2 μg/ml), necrostatin-1 (20 μΜ), necrostatin-5 (20 μΜ), z-VAD-fmk (10 μΜ), 3-MA (10 mM). Cells were rinsed with cold PBS, harvested by scraping into PBS and isolated by centrifugation at 700 × *g* for 5 min, and cell pellets were stored at −80 °C until processing. Cell transfection was performed with Lipofectamine RNAiMAX (Invitrogen) or HiPerfect (Qiagen) according to the protocol provided by the manufacturer. Primers for siRNAs and sgRNAs were listed in Supplementary Table 1. GSDMD KO cells were established as previously described [33].

### Immunofluorescence staining

Cells were grown to 70–80% confluence in 6-well dishes with coverslides, treated with necrocide 1 for 24 h and were fixed with 4% paraformaldehyde solution in PBS at room temperature for 20 min. After permeabilization with PBS buffer containing 0.1% Triton X-100 at room temperature for 20 min, cells were incubated with primary antibodies (anti-cytochrome c, or anti-cathepsin L) at 37 °C for 2 h. After washing with PBS buffer containing 0.1% Triton X-100, cells were incubated with Rhodamine red conjugated secondary antibodies at 37 °C for 2 h. Slides were examined by using a laser scanning confocal microscope (Zeiss LSM 510 META UV/Vis).

### Transmission electron microscopy analysis

For electron microscopy, cells were fixed with 2% glutaraldehyde in 0.1 M sodium cacodylate buffer (pH 7.2) for 2 h followed by 1% osmium tetroxide in 0.1 M sodium cacodylate buffer (pH 7.2) for 2 h. Samples were enblocked with 0.5% aqueous uranyl acetate overnight and treated by low-temperature dehydration and infiltration with a graded series of Epon/Araldite, which was followed by the embedment in 100% Epon/Araldite. Thin sections (60 nM) were cut and stained with Reynalds lead citrate and analyzed with a FEI Tecnai 12 Transmission electron microscope.

### Viability and cell death assays

Cell viability and dose-response curves were measured by WST-1 proliferation assay (Roche), lactate dehydrogenase assay (LDH cytotoxicity detection kit, Roche) or Cell Titer Glo (Cell Titer Glo Assay Kit, Promega) according to manufacturers’ instructions. Cells were treated at indicated concentrations for 72 h. The IC_50_ [nM] was estimated from the dose-response curves by nonlinear regression using GraphPad Prism. For necrocide 1 induced death in BCL2-overexpressing cells, MCF-7 cells stable transfected with empty vector (MCF-7-neo2) or a vector coding for BCL2 (MCF-7-BCL2-wt) were left untreated or treated with 50 and 500 nM necrocide 1 for 10 h. Viability was determined by counting trypan blue negative cells. For each condition, three randomly chosen fields of 100 cells were counted. Columns, mean from three independent triplicate experiments. Necrotic cell death was analyzed by an Olympus IX microscope in the UV channel. Cells were stained with cell-permeable Hoechst 33342 2.5 μg/ml (Sigma) for 10 min. Survival was determined by counting of trypan blue negative cells. Three randomly chosen fields containing a minimum of 100 cells were counted.

### Establishment of mtDNA-depleted MCF-7 cells

As previously reported [51], to develop partially mtDNA-depleted cell lines, we exposed MCF-7 cells to EtBr (0.2 µg/ml) in DMEM medium supplemented for 8 weeks with pyruvate and uridine, which are shown to be essential for the growth of mtDNA-depleted cells. The mtDNA content from MCF-7 cells cultured with or without EtBr was monitored routinely by amplifying total DNA using the following primers: tRNA-Leu (UUR)-F: CAC CCA AGA ACA GGG TTT GT; tRNA-Leu (UUR)-R: TGG CCA TGG GTA TGT TGT TA; microglobulin-F: TGC TGT CTC CAT GTT TGA TGT ATC T; microglobulin-R: TCT CTG CTC CCC ACC TCT AAG T.

### Measurement of reactive oxygen species

Production of reactive oxygen species was determined using H2DCFDA (H2-DCF, DCF, Invitrogen), MitoSox (Invitrogen), BODIPY 581/591 C11 (Invitrogen) and measured by flowcytometry according to manufactures instructions. Briefly, MCF-7 cells were seeded 5 × 10^4^ cells/well in a 12 well plate. Cells were incubated with drugs in the medium for 2 h at the final indicated concentrations.

### In vivo efficacy studies

The anti-tumor effect in vivo was tested in subcutaneous PC-3 prostate and MCF-7 breast (s.c.) xenograft models. Female NMRI nude mice (4–5 weeks) were obtained from Taconic. After at least 1 week of acclimatizing period, each mouse was injected with 1 × 10^7^ in vitro grown cells washed once with PBS and suspended in 100 ml of PBS + 100 ml matrigel (BD). When the tumor volume was about 200–1000 mm^3^ (as indicated), the mice were randomly divided into two groups: NC1 group and control group. NC1 was formulated in 2% DMSO, 20% HP-b-CD and isotonic saline and given *i.v.* or oral at 10 ml/kg. The PC-3 cells were grown in RPMI + 10% FBS, washed once with PBS and suspended in 100 ml of PBS + 100 ml matrigel (BD). Tumor volume (tv) is determined from perpendicular tumor diameters D1 and D2. Tv = p × ((D1 + D2)/2)3⋅1.6. Treatment started at tumor volumes around 200–400 mm^3^, or up to 1000 mm^3^. The experiments were conducted at TopoTarget Copenhagen, and approved by the Experimental Animal Inspectorate, Danish Ministry of Justice; and repeated at Shanghai Jiaotong University School of Medicine, with all related procedures reviewed and approved by the Institutional Animal Care and Use Committee of Shanghai Jiao Tong University School of Medicine.

### Statistical analyses

All assays were conducted at least three times and reproducible results were obtained. All the data are shown as mean ± s.d. unless stated otherwise. The statistical significance between different groups was analyzed by one-way ANOVA (Prism; GraphPad) unless stated otherwise. Statistical significance was defined as *p < 0.05; **p < 0.01; ***p < 0.001.

## Supplementary information

Supplementary file

Original Data File

Supplementary Movie 1

checklist

## Data Availability

Bulk RNA-Seq data were deposited in the NCBI’s Gene Expression Omnibus (GEO) database (GEO GSE226801).
