# Supplementary material for: Necrocide 1 mediates necrotic cell death and immunogenic response in human cancer cells
Source: Cell Death Dis. 2023 Apr 5;14(4):238. doi: 10.1038/s41419-023-05740-0 (PMC10073102; doi:10.1038/s41419-023-05740-0)
Supplement: Supplementary file 1 — Supplementary file [file 41419_2023_5740_MOESM1_ESM.docx]

**Necrocide 1 Mediates Necrotic Cell Death and Immunogenic Response in Human Cancer Cells**

Jing Zhang^1,* +^, Christina Trojel-Hansen^2,3,* +^, Jianghuang Wang^1,*^, Zili Zhang^1^, Xing Wang^1^, Yuhui Qiao^1^, Huike Jiao^1^, Mickaël Michaud^2,3^, Oliver Kepp^2,3^, Marja Jäättelä^4,5^, Guido Kroemer^2,3,6,^ ^+^ and Qing Zhong^1, +^

^1^Key Laboratory of Cell Differentiation and Apoptosis of National Ministry of Education, Department of Pathophysiology, Shanghai Jiao Tong University School of Medicine, 200025 Shanghai, China；

^2^Equipe labellisée par la Ligue contre le cancer, Université de Paris Cité, Sorbonne Université, INSERM UMR1138, Centre de Recherche des Cordeliers, Paris, France

^3^Metabolomics and Cell Biology Platform, Institut Gustave Roussy, 94805 Villejuif, France

^4^Cell Death and Metabolism, Center for Autophagy, Recycling and Disease (CARD), Danish Cancer Society Research Center (DCRC), DK-2100 Copenhagen, Denmark；

^5^Department of Cellular and Molecular Medicine, Faculty of Health Sciences, University of Copenhagen, DK-2200 Copenhagen, Denmark

^6^Institut du Cancer Paris CARPEM, Department of Biology, Hôpital Européen Georges Pompidou, AP-HP, 75015 Paris, France

* : These authors contributed equally to this work.

^+^ : corresponding authors :

Qing Zhong, qingzhong@shsmu.edu.cn; Guido Kroemer, kroemer@orange.fr; Christina Trojel-Hansen, christina.trojel@gmail.com; Jing Zhang, jingzhang@shsmu.edu.cn

**Running title:** Regulated necrosis and anticancer therapy

**Keywords:** programmed cell death**,** TNF independent，necrosis，mitochondria, immunogenic cell death

**Supplementary Figures**


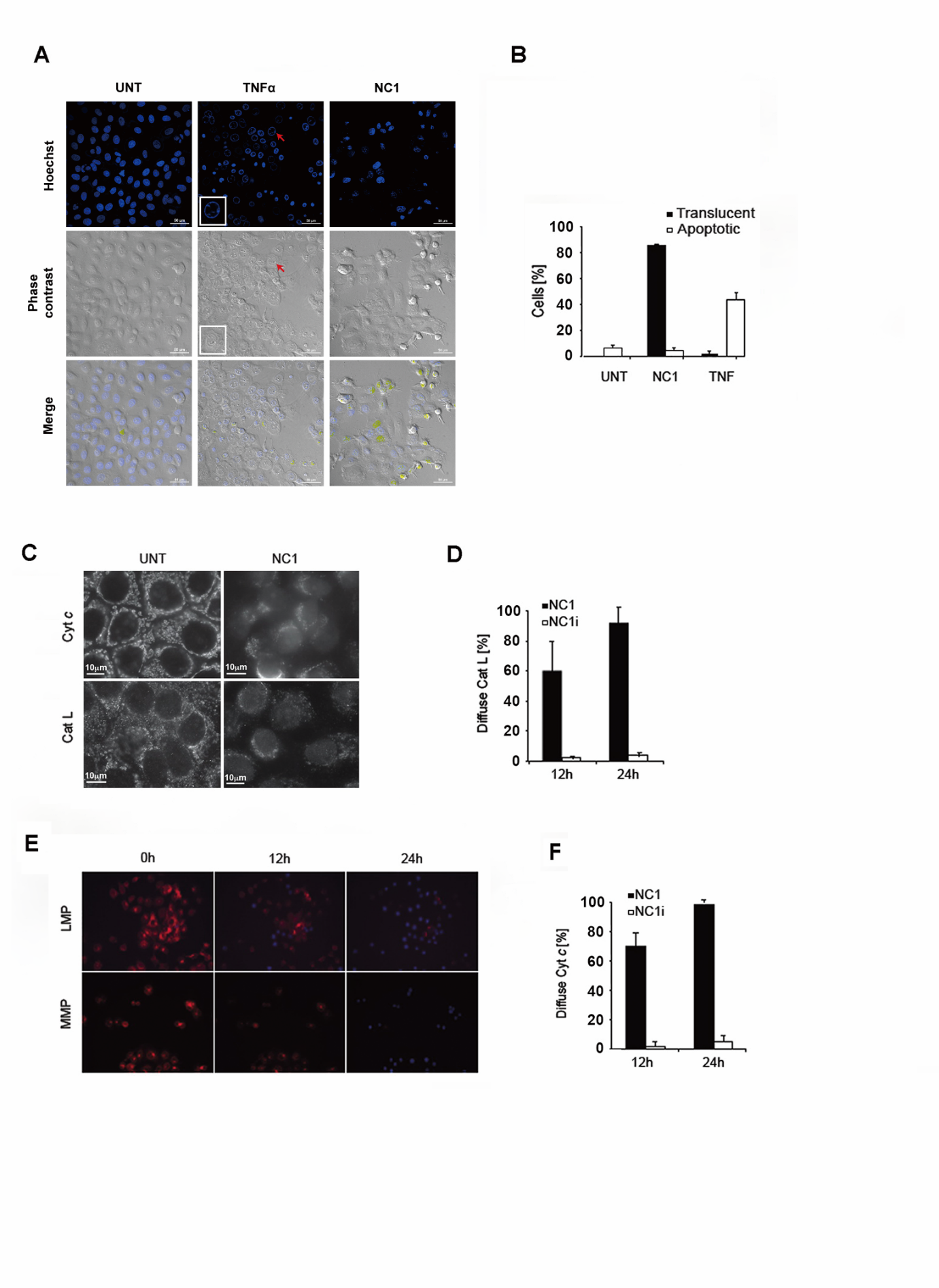


***Supplementary Figure 1.* NC1 induces regulated necrosis in human cancer cells. (A)** NC1-treated or untreated (UNT) MCF-7 cells were stained with Hoechst 33342 to visualize chromatin and examined by fluorescence and phase contrast microscopy (scale bars = 50 µm). (**B**). Percentage of MCF-7 cells exhibiting an apoptotic or non-apoptotic, translucent morphology as shown in A. (**C**). Effects of NC1 on the subcellular localization of cytochrome c and cathepsin L. Cells were stained for the immunofluorescence detection of cytochrome c and cathepsin L. (scale bars = 10 µm). (**D**). Quantitative analysis of cathepsin L release in MCF-7 cells responding to NC1. (**E**). NC1-induced mitochondrial and lysosomal permeabilization. After the indicated incubation period, NC1-treated MCF-7 cells were stained with the Δψm-sensitive dye TMRM or with the lysosomal dye Lysotracker red. Note the loss of punctuate staining patterns visible in representative microphotographs. (**F**). Quantitative analysis of cytochrome c release in MCF-7 cells responding to NC1.


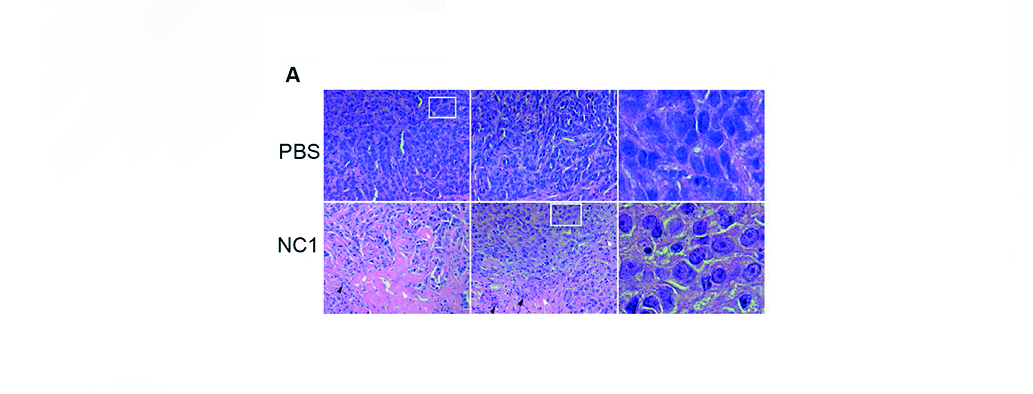
***Supplementary Figure 2.*** **NC1 is a potent antitumor agent *in vivo*. (A)** Tumors (n=20) obtained from mouse *xenografts* of MCF-7 cells that had been given 9 i.v. injections of necrocide 1 at 30 mg/kg (n=10) or saline (n=10) over 21 days were sectioned and stained with hematoxylin and eosin. Note the presence of swollen nuclei that lack a surrounding cytoplasm in NC1-treated tumors. This experiment has been repeated twice, yielding similar results.


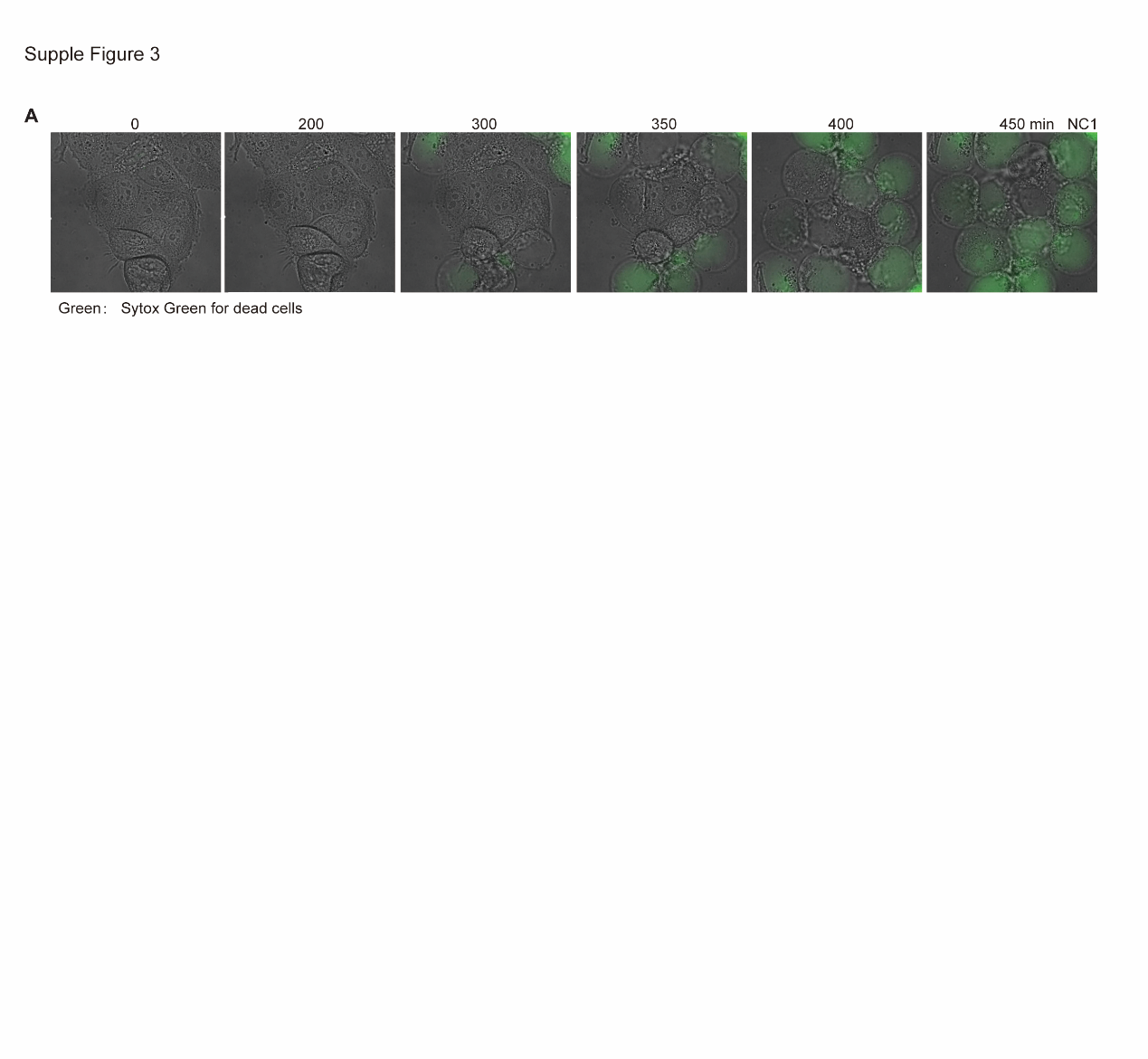


***Supplementary Figure 3.* NC1 elicits a non-apoptotic cell death independent of TNFR1 pathway.** (**A**). MCF-7 cells were treated with NC1 (100 nM) for 8 h and at the same time stained with Sytox Green (1 μM, green signal) to visualize dead cells. Fluorescence and phase contrast images were taken every 10 min after the addition of NC1 and merged images at the indicated times are shown (scale bars = 20 µm).


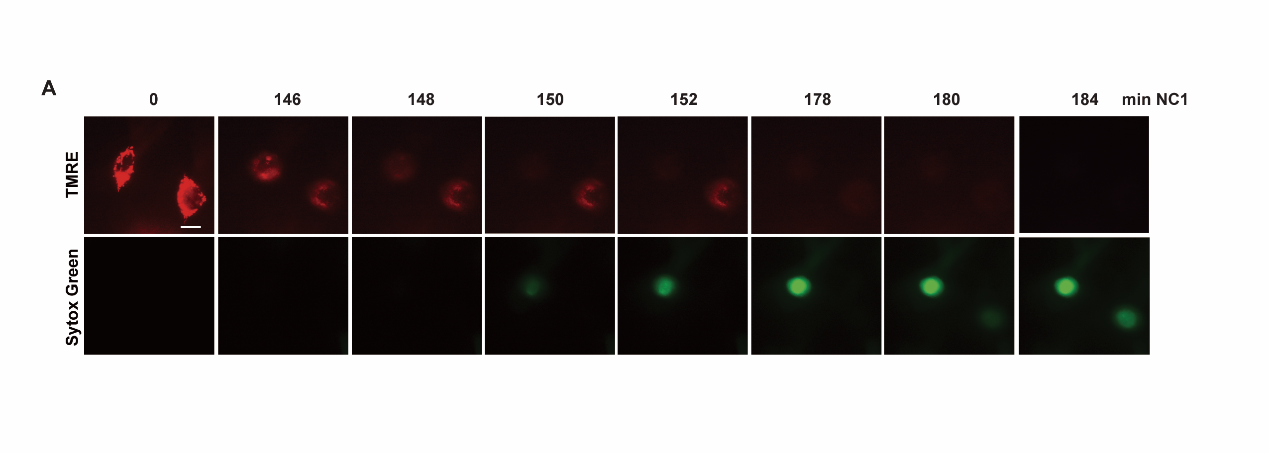


***Supplementary Figure 4. Mitochondria is crucial for NC1-induced cell death.*** (**A**). MCF-7 cells were incubated with TMRE/Sytox-green, treated with NC1, and imaged by live-cell confocal microscopy. Images obtained after initial treatment and at the point of cell lysis are shown (scale bars = 20 µm).

**Supplementary Movie 1**. MCF-7 cells were treated with NC1 (100 nM) for 8 h and at the same time stained with Sytox Green (1 μM, green signal) to visualize dead cells. The bright field and sytox green images were taken every 10 minuts.

***Supplementary Table 1. Primers for siRNA or sgRNA.***

| Gene | oligo | Sequences (5’-3’) |
| --- | --- | --- |
| BAX | siRNA | GGGACGAACTGGACAGTAA |
| BAK | siRNA | CCCATTCACTACAGGTGAA |
| CypD | siRNA-1 | GGACTCTAATACCTGTTTA |
| CypD | siRNA-2 | GATGCGGATATAGATTTAA |
| MLKL | sgRNA | CACACCGTTTGTGGATGACC |
| ACSL4 | sgRNA | GTGAAAGAATACCTGGACTGGGG |
