# Supplementary material for: Necrocide 1 mediates necrotic cell death and immunogenic response in human cancer cells
Source: Cell Death Dis. 2023 Apr 5;14(4):238. doi: 10.1038/s41419-023-05740-0 (PMC10073102; doi:10.1038/s41419-023-05740-0)
Supplement: Supplementary file 2 — Original Data File [file 41419_2023_5740_MOESM2_ESM.pptx]

## Slide 1
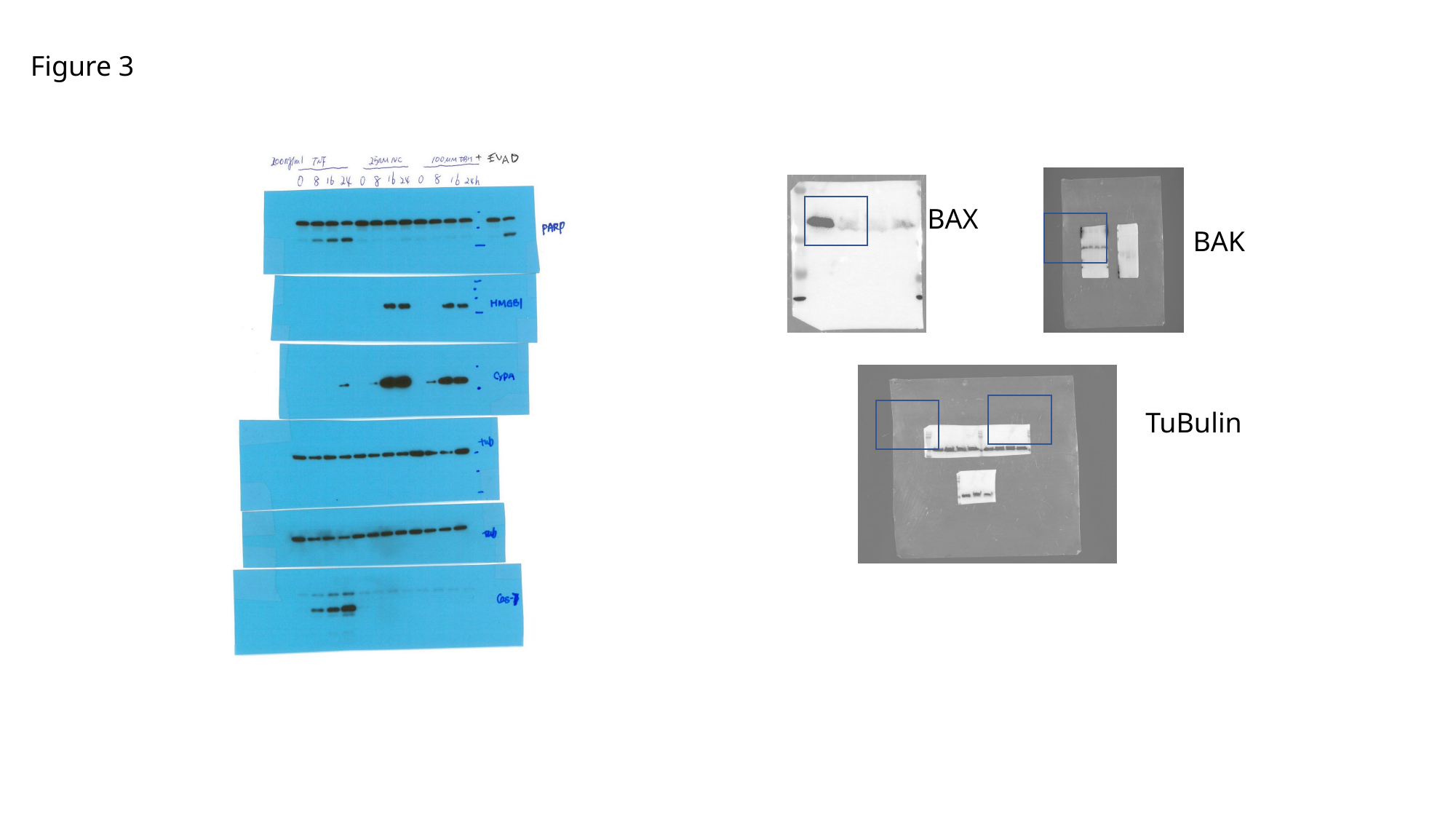

Figure 3
BAX
BAK
TuBulin

## Slide 2
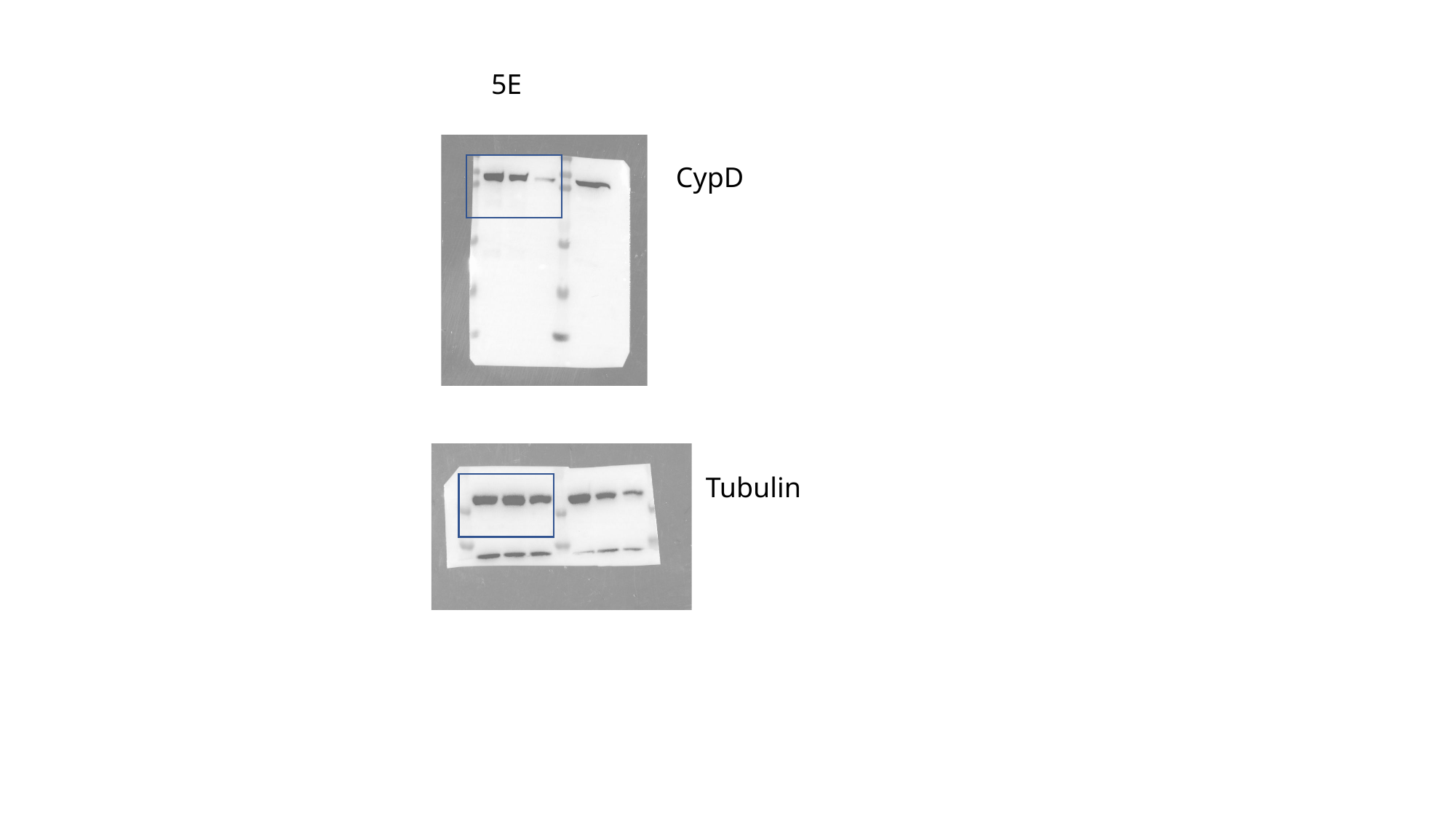

5E
CypD
Tubulin

## Slide 3
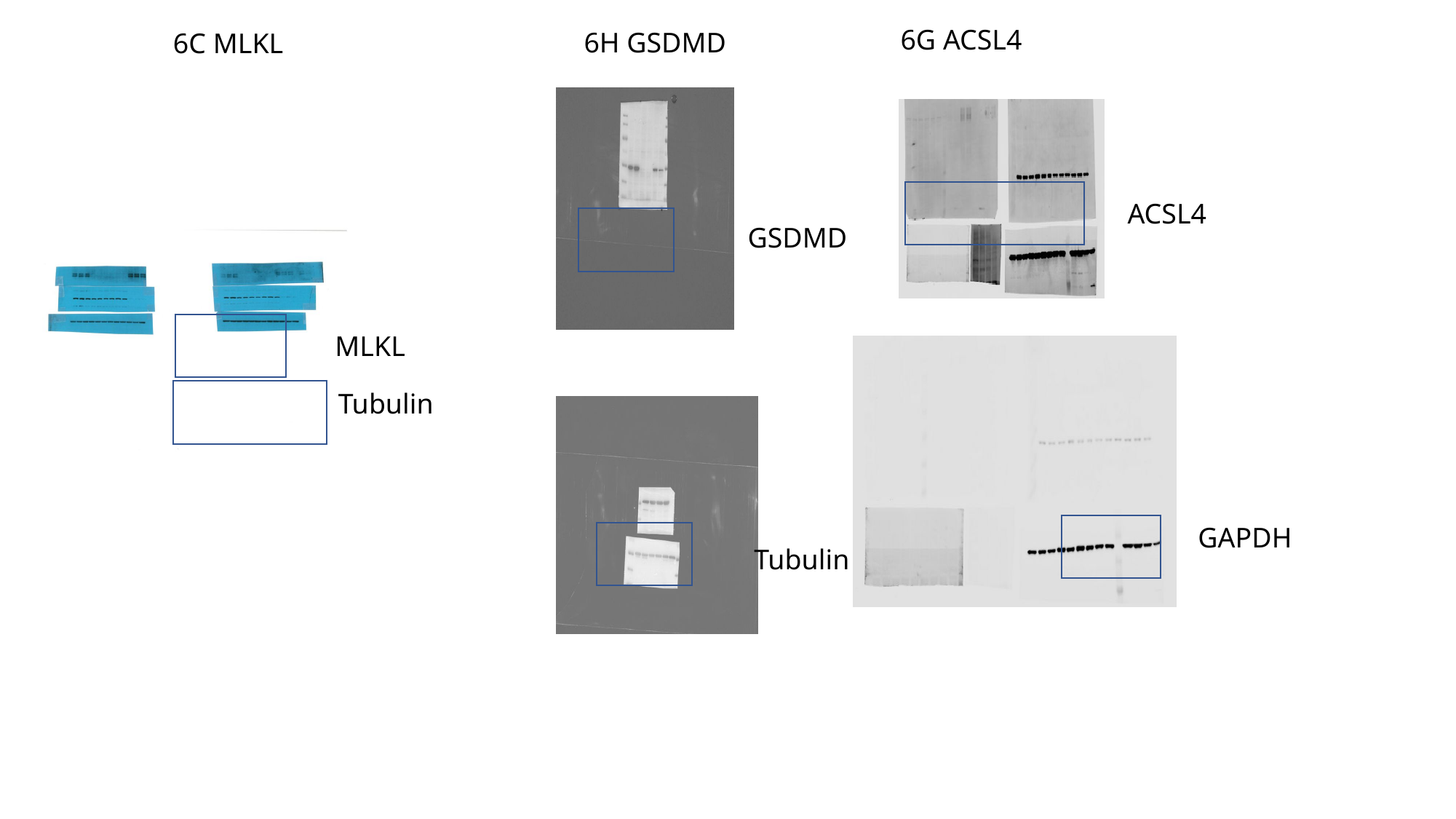

6G ACSL4
6H GSDMD
6C MLKL
ACSL4
GSDMD
MLKL
Tubulin
GAPDH
Tubulin

## Slide 4
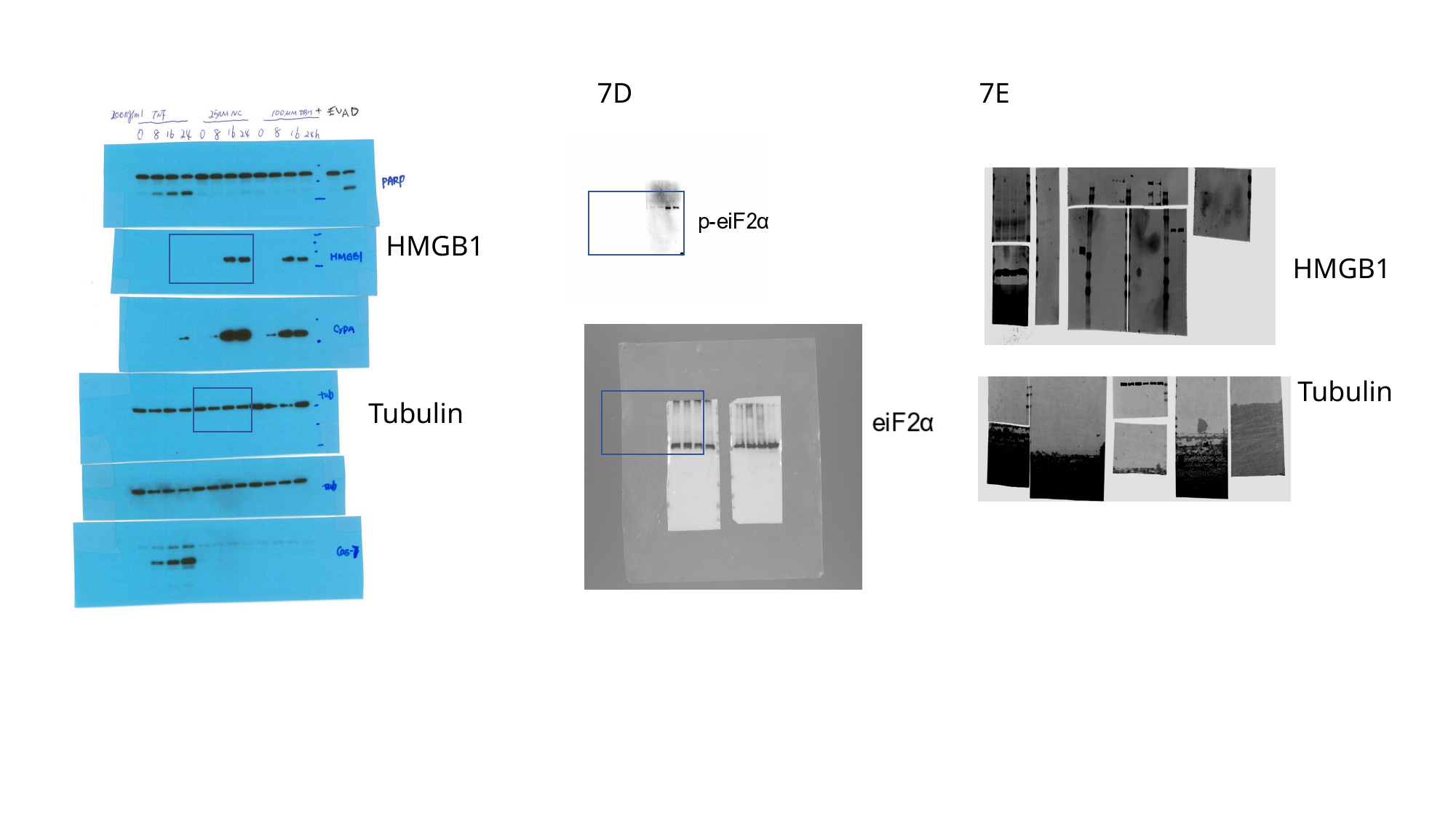

7D
7E
HMGB1
HMGB1
Tubulin
Tubulin
